# MMP-11 promoted the oral cancer migration and FAK/Src activation

**DOI:** 10.18632/oncotarget.15824

**Published:** 2017-03-02

**Authors:** Chung-Han Hsin, Ying-Erh Chou, Shun-Fa Yang, Shih-Chi Su, Yi-Ting Chuang, Shu-Hui Lin, Chiao-Wen Lin

**Affiliations:** ^1^ School of Medicine, Chung Shan Medical University, Taichung, Taiwan; ^2^ Department of Otolaryngology, Chung Shan Medical University Hospital, Taichung, Taiwan; ^3^ Department of Medical Research, Chung Shan Medical University Hospital, Taichung, Taiwan; ^4^ Institute of Medicine, Chung Shan Medical University, Taichung, Taiwan; ^5^ Whole-Genome Research Core Laboratory of Human Diseases, Chang Gung Memorial Hospital, Keelung, Taiwan; ^6^ Department of Surgical Pathology, Changhua Christian Hospital, Changhua, Taiwan; ^7^ Institute of Oral Sciences, Chung Shan Medical University, Taichung, Taiwan; ^8^ Department of Dentistry, Chung Shan Medical University Hospital, Taichung, Taiwan

**Keywords:** matrix metalloproteinase, oral squamous cell carcinoma, metastasis, survival, FAK/Src pathway

## Abstract

Matrix metalloproteinase-11 (MMP-11) has been observed in most invasive human carcinomas. The current study investigated the association between the clinicopathological characteristics and MMP-11 expression in oral squamous cell carcinoma (OSCC) patients. Immunohistochemistry (IHC) staining was performed to assess MMP-11 expression in 279 patients with OSCC. In addition, the metastatic effects of the MMP-11 overexpression on the OSCC cells were also investigated. We found that MMP-11 expression was present in 118/279 (42.3%) cases and expression of MMP-11 was associated with higher incidence of lymph node metastasis and worse grade of tumor differentiation. Importantly, OSCC patients with strong expression of MMP-11 had a significantly lower survival rate (p=0.010). Furthermore, MMP-11 overexpression in OSCC cells increased *in vitro* cell migration. Mechanistically, MMP-11 increased the cell motility of OSCC cells through focal adhesion kinase/Src kinase (FAK/Src) pathway. In conclusion, our results revealed that the MMP-11 expression in OSCC samples can predict the progression, especially lymph node metastasis, and the survival of OSCC patients in Taiwan.

## INTRODUCTION

Oral squamous cell carcinoma (OSCC) is the most common malignancy of the head and neck worldwide and, in some Asian countries such as India and Taiwan, may accounting for more than 10% of all malignancies [1, 2]. Prior studies have shown evidences that the susceptibility to OSCC of an individual is strongly mediated by some carcinogen-exposure behaviors, such as betel nut [3, 4]. Traditionally, the mainstay treatment for OSCC includes excision of the primary tumor, with or without dissection of the neck lymph nodes. For patients with advanced diseases or pathologic risk factors, adjuvant radiotherapy and/or chemotherapy are also parts of the standard treatments [5]. Despite ongoing advances in the surgical techniques and adjuvant therapies, the 5-year overall survival rate remains unfavorable in a considerable portion of OSCC patients because invasion of the neighboring tissues and metastasis to the neck lymph nodes are common [6, 7]. Therefore, identifying new biomarkers that can predict the risk of OSCC progression, especially local invasion and lymph node metastasis, is mandatory to improve the treatment of this deadly disease.

For cancers to invade and metastasize, tumor cells must degrade the extracellular matrix (ECM) and gain access to blood vessels and lymphatics [8]. Matrix metalloproteinases (MMPs) are zinc-dependent endopeptidases that can degrade the components of the ECM and basement membranes, and increased expression of these enzymes is detected in almost all human cancers and has been associated with the aggressiveness in various neoplasms [8–11]. MMP-11, also known as stromelysin-3, is closely related with tissue remodeling during involution, embryogenesis, and wound healing in normal physiologic conditions [12]. Like many other members of MMPs, increased expression of MMP-11 has been observed in most invasive human carcinomas, including lung, breast, colorectal and ovarian carcinomas, and high levels of its mRNA was reportedly associated with aggressive phenotypes and poor clinical outcome [13–15].

The clinical significance of MMP-11 expression has been demonstrated previously by immunohistochemical studies on tumor specimens of OSCC patients. Soni et al found that, in tobacco-associated OSCC, expression of MMP-11 was significantly associated with the involvement of neck lymph node [16]. The data, however, failed to demonstrate the clinical significance of MMP-11 expression in OSCC tissues which are closely related to the consumption of betel nut. To address this issue, and also to determine whether measurement of MMP-11 expression could possibly serve as a prognostic indicator for OSCC, we conducted an immunohistochemical analysis to investigate the relationships between the expression of MMP-11 and clinicopathologic parameters in 279 patients with OSCC. Moreover, in established human oral cancer cell lines, we further evaluated the migration capability in MMP-11 overexpressed OSCC cell lines and the underlying mechanisms.

## RESULTS

### Patient characteristics

The demographic and clinicopathological data of this study subjects were shown in Table 1. A total of 279 patients (264 men, 15 women) with OSCC were included in the current investigation. The patients were aged 31 to 90 years (mean age=55.80 ± 11.15 years). The tumors located over the following sites: buccal mucosa (n=109), tongue (n=93), gingiva (n=34), palate (n=16) and floor of the mouth (n=14). As to TNM staging of the OSCC, 53 (19.0 %) were at stage I, 56 (20.1 %) were at stage II, 35 (12.5 %) were at stage III, and 135 (48.4 %) were at stage IV. Grade of tumor cell differentiation included 42 (15.1%) well differentiation and 237 (84.9%) moderate or poor differentiation.

**Table 1:** Distributions of demographical characteristics in 279 patients with oral cancer

Characteristics	Total (%)
**Total number of patients**	**279**
**Age (year)**	
Mean ± SD	55.80 ± 11.15
**Gender**	
Male	264 (94.6%)
Female	15 (5.4%)
**Cancer location**	
Buccal mucosa	109 (39.1 %)
Tongue	93 (33.3 %)
Gingiva	34 (12.2 %)
Palate	16 (5.7 %)
Floor of Mouth	14 (5.0 %)
Others	13 (4.7 %)
**Clinical stage**	
I	53 (19.0%)
II	56 (20.1%)
III	35 (12.5%)
IV	135 (48.4%)
**T classification**	
T1	70 (25.1%)
T2	88 (31.5%)
T3	23 (8.2%)
T4	98 (35.1%)
**N classification**	
N0	176 (63.1%)
N1	36 (12.9%)
N2	63 (22.6%)
N3	4 (1.4%)
**M classification**s	
M0	276 (98.9%)
M1	3 (1.1%)
**Grade**	
Well	42 (15.1%)
moderate, poor	237 (84.9%)

### Association of expression of MMP-11 and clinicopathological characteristics in OSCC

**Figure 1:**
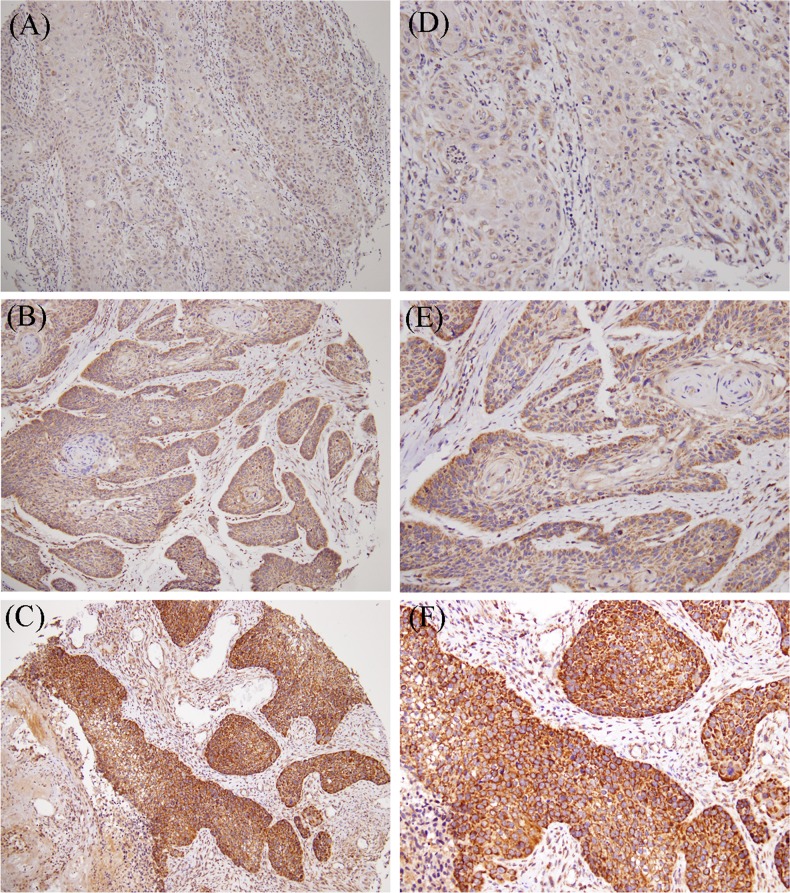
MMP-11 expression in primary oral cancer Tissue microarrays of primary oral squamous cell carcinomas (OSCCs) were immunohistochemically analyzed for MMP-11. **(A and D)** no detectable MMP-11 (0). **(B and E)** weak expression levels (1+). **(C and F)** strong expression levels (2+). **(A-C)** low-power field (100x); **(D-F)** high-power field (200x).

According to the expression level in the OSCC tissues (Figure 1), we divided MMP-11 immunohistological stains into two groups: weak and strong. Weak expression of MMP-11 was present in 161 out of 279 patients (57.7 %), while strong expression in 118 patients (42.3 %). Statistical analysis revealed no significant differences between MMP-11 expression and age, gender, cancer location, clinical stage, tumor classification and distant metastasis. Patients with strong MMP-11 expression, however, were associated with higher incidence of lymph node metastasis (p=0.034) and worse grade of tumor differentiation (p=0.009) (Table 2).

**Table 2:** Patient characteristics regarding MMP-11 expression

Characteristics	No. of patients (%)		
	MMP-11 (weak)	MMP-11 (strong)	p value
Total number of patients	161 (57.7)	118 (42.3)	
**Age (year)**			
<55	84 (52.2)	56 (47.5)	0.436
≥55	77 (47.8)	62 (52.5)	
**Gender**			
Male	155 (96.3)	109 (92.4)	0.154
Female	6 (3.7)	9 (7.6)	
**Cancer location**			
Buccal mucosa	71 (44.1)	38 (32.2)	0.164
Tongue	52 (32.3)	41 (34.7)	
Gingiva	18 (11.2)	16 (13.6)	
Others	20 (12.4)	23 (19.5)	
**Clinical stage**			
I+II	64 (39.8)	45 (38.1)	0.785
III+IV	97 (60.2)	71 (61.9)	
**T classification**			
T1+T2	88 (54.7)	70 (59.3)	0.437
T3+T4	73 (45.3)	48 (40.7)	
**N classification**			
N0	110 (68.3)	66 (55.9)	0.034*
N1+2+3	51 (31.7)	52 (44.1)	
**M classification**			
M0	159 (98.8)	117 (99.2)	0.752
M1	2 (1.2)	1 (0.8)	
**Grade**			
Well	32 (19.9)	10 (8.5)	0.009*
moderate, poor	129 (80.1)	108 (91.5)	

* p value < 0.05 as statistically significant.

### Survival analyses of MMP-11 and clinicopathological parameters

Univariate analysis using Cox proportional hazards regression model showed that advanced clinical stage (p< 0.001), large tumor size (p=0.002), positive lymph node metastasis (p< 0.001), worse grade of differentiation (p=0.027) and strong expression of MMP-11 (p=0.010) were correlated with a poor overall survival of patients with OSCC (Table 3). However, no significant association was found between age, gender and survival rate (Table 3). If analyzed with multivariate analysis using Cox regression model, the results showed that T status (p=0.024), N status (p< 0.001) and MMP-11 expression (p=0.043) were correlated with a poor overall survival of patients with OSCC, while there was no such association for clinical stage and differentiation grade with the prognosis of OSCC patients (Table 4).

**Table 3:** Univariate survival analyses of MMP-11 and clinicopathological parameters among patients with OSCC using the Cox proportional hazard regression model

All cases (N=279)	Hazard ratio (95% CI)	p value
Age (<55 versus ≥55)	0.987 (0.615–1.585)	0.958
Gender (Male versus Female)	1.504 (0.530–4.268)	0.443
Clinical stage (stage I + II versus stage III + IV)	2.929 (1.780–4.818)	< 0.001*
T status (T1 + T2 versus T3 + T4)	2.185 (1.337–3.569)	0.002*
N status (N0 versus N1 + N2 + N3)	4.136 (2.398–7.133)	< 0.001*
Grade (Well versus moderate, poor)	2.122 (1.088–4.141)	0.027*
MMP-11 (weak versus strong)	1.902 (1.166–3.100)	0.010*

* p value < 0.05 as statistically significant.

**Table 4:** Multivariate survival analyses of MMP-11 and clinicopathological parameters among patients with OSCC using the Cox regression model

All cases (N=279)	Hazard ratio (95% CI)	p value
Clinical stage (stage I + II versus stage III + IV)	0.601 (0.202–1.788)	0.360
T status (T1 + T2 versus T3 + T4)	2.878 (1.150–7.204)	0.024*
N status (N0 versus N1 + N2 + N3)	4.655 (1.973–10.984)	< 0.001*
Grade (Well versus moderate, poor)	1.662 (0.813–3.397)	0.164
MMP-11 (weak versus strong)	1.726 (1.017–2.930)	0.043*

* p value < 0.05 as statistically significant.

**Figure 2:**
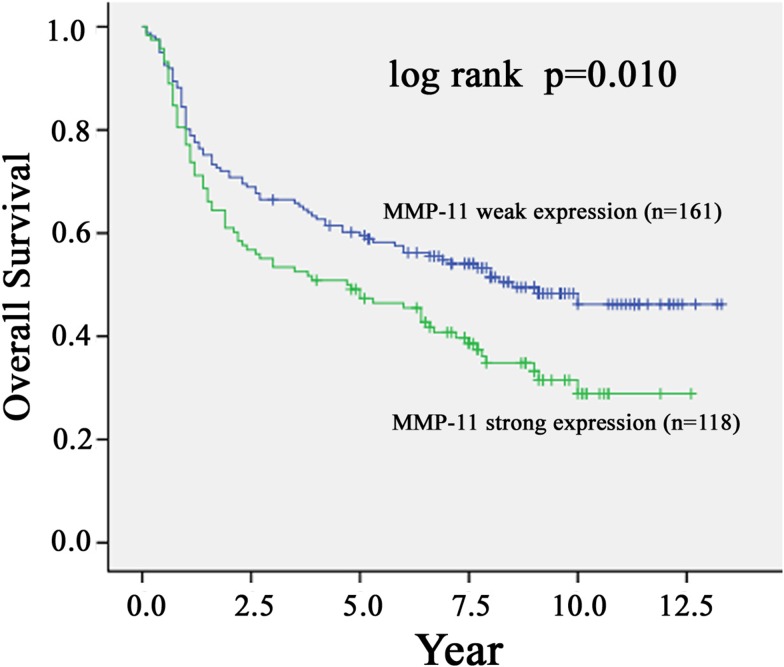
Kaplan-Meier survival curve showing the relation between MMP-11 expression in primary tumors and survival in 279 oral squamous cell carcinoma (OSCC) patients The overall survival of OSCC patients with positive MMP-11 staining was significantly lower than that of OSCC patients with negative MMP-11 staining (p<0.05, log-rank test).

A Kaplan-Meier analysis was also performed to evaluate the association between the expression of MMP-11 and overall survival. The analysis showed that OSCC patients with strong expression of MMP-11 had a significantly lower survival rate (p=0.010) (Figure 2). The median survival in weak expression of MMP-11 was 79.2 months, whereas that in strong expression of MMP-11 was 48 months.

### MMP-11 promoted the cell migration of OSCC cell lines

**Figure 3:**
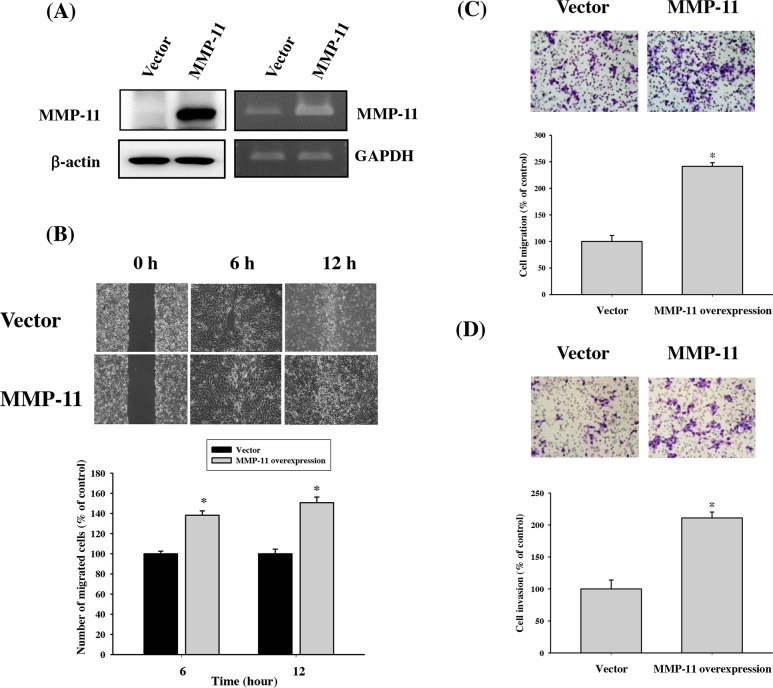
The relationships between MMP-11 expression and cell migration in TW2.6 OSCC cell lines **(A)** The MMP-11 expression of TW2.6 cells transfected with pcDNA3.0-MMP-11 was examined by western blot and RT-PCR. **(B-D)** Detection of cell migratory abilities by transfection with MMP-11 overexpression vector in TW2.6 cell. Migratory abilities of pcDNA3.0 and pcDNA3.0-MMP-11 cells were evaluated using **(B)** wound healing assay. The **(C)** migratory and **(D)** invasive abilities of pcDNA3.0 and pcDNA3.0-MMP-11 cells were evaluated using Boyden chamber migration and Matrigel invasion assays. Differences are presented as the mean of triplicate experiments compared with control cells. *p < 0.05 compared with control cells.

Since we found that expression of MMP-11 was significantly correlated with the presence of lymph node metastasis, the effects of the MMP-11-overexpression on the OSCC cell line were investigated by *in vitro* wound-closure assay, Boyden chamber cell invasion and migration assay. The MMP-11 expression of TW2.6 cells transfected with pcDNA3.0-MMP-11 was confirmed by western blot and RT-PCR (Figure 3A). We next evaluated the effect of MMP-11 overexpression on TW2.6 cell migration. Using an *in vitro* wound-closure assay, Boyden chamber cell migration assay and invasion assay, it was shown that MMP-11 overexpression significantly increased the migration capability of TW2.6 cells (Figure 3B-3D).

### Effects of MMP-11 expression on regulation of focal adhesion kinase/Src kinase (FAK/Src) and MAP-Kinase signalling pathway

**Figure 4:**
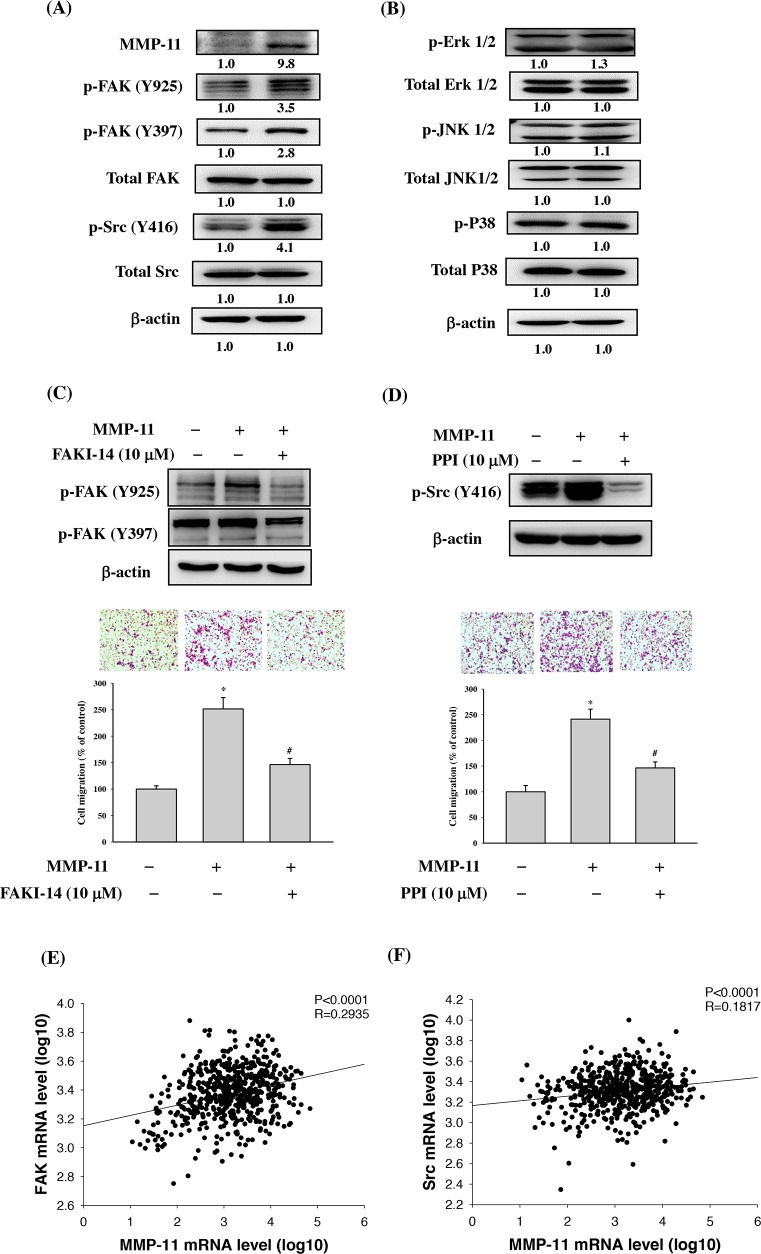
FAK/Src signaling pathway is involved in MMP-11-promoted cell migration **(A)** TW2.6 cells transfected with pcDNA3.0 and pcDNA3.0-MMP-11 overexpression vector for 24 h and the total cell lysates were then subjected to western blot to analyze the phosphorylation of **(A)** FAK and Src **(B)** Erk 1/2, JNK1/2 and p38 as described in the Materials and Methods section. **(C)** TW2.6 cells were treated with a FAK inhibitor (FAKI-14; 10μM) for 24h. FAK phosphorylation was examined by western blot and cell migration was examined by Boyden chamber migration. **(D)** TW2.6 cells were treated with a Src inhibitor (PPI; 10μM) for 24h. Src phosphorylation was examined by western blot and cell migration was examined by Boyden chamber migration. Data are expressed as the mean ± SEM *p < 0.05 compared with control; #p < 0.05 compared with the pcDNA3.0-MMP-11 overexpression vector group. **(E-F)** The correlations among mRNA levels of MMP-11 and FAK as well as Src in head and neck squamous cell carcinoma from The Cancer Genome Atlas (TCGA) Data Portal. **(E)** A significant correlation was found between MMP-11 and FAK (Spearman rank correlation coefficient r =0.2935, p<0.0001). **(F)** A significant correlation was found between MMP-11 and Src (Spearman rank correlation coefficient r =0.1817, p<0.0001).

The focal adhesion kinase/Src kinase (FAK/Src) and Mitogen-activated protein kinases (MAPK) pathway is known to play a role in cancer metastasis [17–21]. To elucidate the involvement of FAK/Src and MAPK signaling in MMP-11 overexpression TW2.6 cell, western blot analysis was performed. The results revealed phosphorylation of FAK and Src were significantly increased in the MMP-11-overexpressed TW2.6 OSCC cells compared with those in their parental, whereas it had no effect on ERK1/2, JNK1/2 and p38 phosphorylation (Figures 4A-4B). Moreover, pretreatment with FAK inhibitor (FAKI-14) or Src inhibitor (PPI) reversed the MMP-11 overexpression-induced migration in OSCC cells (Figure 4C and 4D). These results indicate that MMP-11 increases OSCC migration may through the FAK/Src signaling pathway. To further support this conclusion, we also examined the expression of MMP-11, FAK and Src in HNSCC tissue by using the The Cancer Genome Atlas (TCGA) Database. The results showed that the relative MMP-11 levels were positively correlated with the expression of FAK (Spearman rank correlation coefficient r =0.2935, p<0.0001) and Src (Spearman rank correlation coefficient r =0.1817, p<0.0001) mRNAs in HNSCC (Figures 4E-4F).

## DISCUSSION

Tobacco use and alcohol consumption are well-known carcinogen-exposure behaviors and risk factors for most head and neck cancers, especially OSCC [22]. Betel nut chewing, a very common social behavior in Taiwan, is another well-established etiological factor in the tumorigenesis of OSCC [3, 23]. For these patients, oral tumors develop most frequently on the tongue and the buccal mucosa, where most contact and irritation by the betel nut occur [24]. The high prevalence of OSCC, ongoing increase of betel nut chewing, and grave outcomes for advanced OSCC, have become an important healthcare issue in Taiwan. In the current study, we examined the correlation between clinicopathological characteristics and MMP-11 expression in OSCC patients, most of them had the behaviors of tobacco use and/or betel nut chewing. Our data showed that a strong expression of MMP-11 was associated with increased lymph node metastasis and poor survival in these patients.

Most members of MMPs have been implicated in the tumorigenesis of various human malignancies. For head and neck cancers, MMP-1, MMP-2, MMP-9 and membrane type-1 MMP were constantly observed overexpressed in the tumor tissues and were considered associated with the development and progression of cancer [10, 25–29]. Expression of MMP-11 in the head and neck cancers, on the other hand, was scarcely investigated in the literature. Soni et al performed immunohistochemical analysis of MMP-11 expression in tumor specimens of 177 OSCC patients and found positive expression in 70% of these samples [16]. Their data also demonstrated that MMP-11 positivity was correlated with metastasis of neck lymph node, although survival analysis failed to reveal association with the expression of MMP-11. Another study of serial paraffin sections, which included 220 OSCC and 90 precancerous lesions, discovered that concomitant expression of MMP-11 and proangiogenic factors was an indicator for progression from precancerous stage to flank malignancy [22]. Of note, study subjects of the abovementioned researches were mainly tobacco and/or alcohol-associated OSCC, while our study enrolled betel nut-associated OSCC as well in Taiwan. Unlike the results of Soni’s study, our data showed that strong expression of MMP-11 was correlated with a poor overall survival of OSCC patients, suggesting a relatively important role of MMP-11 in the aggressiveness of OSCC in Taiwan.

The FAK and Src activation is capable of modulating cell migration and invasion [18, 19, 30]. Our previous study revealed that FAK phosphorylation was involved in caffeic acid phenethyl ester-inhibited oral cancer cell metastasis [21] and WISP1-induced OSCC angiogenesis [31]. Other studies have also reported that FAK/Src pathway was associated the OSCC cell migration [32, 33]. Moreover, Pal et al. shows that thrombospondin-1 promotes migration of oral cancer cells and stimulates the expression of MMP-11 partly through the integrin signaling [34]. Therefore, we utilized a western blot to characterize the phosphorylation of FAK and Src in the MMP-11-overexpressed TW2.6 OSCC cells and showed that phosphorylation of FAK and Src was significantly increased. These data suggested that FAK/Src signaling is involved in MMP-11-mediated cell metastasis in OSCC. However, limitations do exist in our investigation and should be addressed. The present study was the lacking of *in vivo* animal study, which could provide additional support to our findings and will be included in our future work.

In summary, by using immunohistochemical analysis, we observed that strong expression of MMP-11 in OSCC tissues was associated with an increased incidence of lymph node metastasis. In addition, strong MMP-11 expression was significantly related to poor overall survival in patients with OSCC. The results suggest that MMP-11 is a potential biomarker for prognostic indicator in patients with OSCC.

## MATERIALS AND METHODS

### Patients and tissue microarrays

We constructed formalin-fixed, paraffin-embedded tissue microarrays composed of 279 OSCC tissue cores as previously described [35]. Diagnosis of OSCC was based on histological examination of hematoxylin and eosin-stained tissue sections. Approval from the Institutional Review Board of Chung-Shan Medical University Hospital and Changhua Christian Hospital was obtained prior to this study.

### Immunohistochemical (IHC) analysis

Paraffin-embedded OSCC tissue sections (4 μm) of the paraffin slice on coated slides were washed with xylene to remove the paraffin as previously described [36]. After incubation with an anti-MMP-11 (1:100 dilution; Santa Cruz Biotechnology, Santa Cruz, CA) antibody for 60 min at room temperature, slides were thoroughly washed three times with PBS. The conventional streptavidin peroxidase method (LSAB Kit K675; DAKO, Copenhagen, Denmark) was performed for signal development. The intensity of staining was respectively scored −, 1+, and 2+ for weak (− or 1+) and strong staining (2+). All immunohistochemical staining cases were examined by two pathologists and a final agreement was obtained for each score at a discussion microscope.

### Cell culture and full-length MMP-11 plasmid DNA transfection

TW2.6 cells, derived from a buccal cancer patient who chewed Betel quid [37], were cultured in Dulbecco’s modified Eagle’s medium supplemented with an equal volume of a nutrient mixture, F-12 Ham’s medium (Life Technologies, Grand Island, NY, USA). All cell cultures were maintained at 37°C in a humidified atmosphere of 5% CO_2_. The pcDNA3.0-MMP-11 expression vector and pcDNA3.0 control vector were transiently transfected into TW2.6 cells using Lipofectamine 2000 (Invitrogen, Carlsbad, CA, USA). The MMP-11-overexpressing TW2.6 cells were established and the MMP-11 expression levels shown by these cells were confirmed using western blot and RT-PCR analysis.

### *In vitro* wound closure

TW2.6 cells (5 × 10^4^ cells/well) were plated in 6 cm petri dishes for 24 h and wounds were produced by manually scratching with a 200 μL pipette tip as described previously [7]. Images were recorded at indicated times after treatment using a phase-contrast microscope (×100).

### Cell migration and invasion assays

After a treatment with the MMP-11 overexpression for 24 h, TW2.6 cells were harvested and seeded to Boyden chamber (Neuro Probe, Cabin John, MD, USA) at 10^4^ cell/well in serum free medium and then incubated for 24 h at 37°C. The invaded cells were fixed with 100% methanol and stained with 5% Giemsa. Cell numbers were counted under a light microscope as previously described [38]. To determine cell Invasion, the cells were seeded into the Boyden chamber on membrane filters that were coated with Matrigel [39].

### Western blot analysis

Cellular lysates were prepared by suspending 2×10^6^/10cm dish in 200μL of RIPA buffer containing protease inhibitors cocktail. Cell lysates were subjected to a centrifugation of 10,000 rpm for 10 min at 4°C, and the insoluble pellet was discarded. The 20 μg samples of cell lysates was separated by SDS-PAGE on 10% polyacrylamide gels and transferred onto a nitrocellulose membrane using the Mini-Protean Tetra Electrophoresis System as described previously [18]. The blot was subsequently incubated with 5% non-fat milk in Tris-buffered saline (20 mM Tris, 137 mM NaCl, pH 7.6) for 1 h to block non-specific binding and then overnight with polyclonal antibodies against MMP-11 (Santa Cruz, CA, USA) and β-actin (Novus Biologicals, Co, USA). Blots were then incubated with a horseradish peroxidase anti-rabbit IgG for 1 h. Afterwards, signal was detected by using enhanced chemiluminescence (ECL) commercial kit (Amersham Biosciences, Piscataway, NJ, USA).

### Reverse transcriptase–polymerase chain reaction (RT-PCR)

Total RNA was isolated using Total RNA mini kit (Qiagen, Valencia, CA, USA) and reverse transcribe into cDNA using High Capacity cDNA Reverse Transcription kit (Applied Biosystems, Foster City, CA, USA) as previously described [40]. The sequences of the primers were: F-5′-CAGGTGGCAGCCCATGAATT3′and R-5′- GTACTGAGCACCTTGGAAGA-3′ for MMP-11, and F-5′-CGGAGTCAACGGATTTGGTCGTAT-3′ and R-5′-AGCCTTCTCCATGGTTGGTGAAGAC-3′ for GAPDH. The reaction mix was first denatured at 95°C for 5 min. The PCR condition for MMP-11 were 94°C for 1 min, 64°C for 1 min, 72°C for 2 min for 30 cycles; for GAPDH were 94°C for 1 min, 65°C for 1 min, 72°C for 2 min for 18 cycles, followed by 72°C for 10 min.

### Statistical analysis

Correlations of MMP-11 with clinicopathologic parameters of OSCC were examined by Pearson’s χ^2^ test or Fisher exact test. Cumulative survival was analyzed by the Kaplan-Meier method. Univariate analysis was analyzed by the Cox proportional hazards regression model and multivariable analysis using a Cox regression model (SPSS, Version 17.0; SPSS, Inc, Chicago, IL, USA). Difference between control and treated groups were calculated by Student’s *t*-test and p < 0.05 was considered as statistically significant.
